# Primary endobronchial Hodgkin's disease

**DOI:** 10.4103/0970-2113.56350

**Published:** 2009

**Authors:** Prakash R. Malur, Gajanan S. Gaude, Hema B. Bannur, Shivappa B. Anurshetru, Vijayalaxmi V. Suranagi, Ranjit P. Kangle, Annasaheb J. Dhumale, Pradeep H. Patil, Reshma Davanagere

**Affiliations:** *Department of Pathology, J. N. Medical College, Belgaum, India*; 1 *Department of Respiratory Medicine, J. N. Medical College, Belgaum, India*; 2 *Department of Cardio-Thoracic Vascular Surgery, J. N. Medical College, Belgaum, India*; 3 *Department of Medicine and Medical Oncology, J. N. Medical College, Belgaum, India*; 4 *Department of Radiology, J. N. Medical College, Belgaum, India*

**Keywords:** Endobronchial, primary, pulmonary Hodgkin's disease

## Abstract

We report a case of primary pulmonary Hodgkin's disease presenting as an endobronchial mass. Tissue diagnosis was made by microscopic examination following open thoracotomy and excision biopsy of the mass. The patient responded well to the chemotherapy regimen.

## INTRODUCTION

Pulmonary involvement in Hodgkin's disease is seen in 15% to 40% of patients at some time during the course of illness. However, Hodgkin's disease presenting as pulmonary disease is rare.[1] It presents as a solitary mass, multinodular disease or a cavitatory lesion, in the sixth or seventh decade of life. While the condition often disseminates, its limitation to lung offers a chance for early treatment and good prognosis. A case of primary Hodgkin's disease presenting as an endobronchial mass and showing good response to treatment is reported here.

## CASE REPORT

A 43-year-old woman presented with dry cough, breathlessness (grade III), and chest pain of two months' duration. On examination, her vital signs were normal. There was no peripheral lymphadenopathy or clubbing. Respiratory system examination showed reduced breath sounds on the left side. There was no hepatosplenomegaly. Laboratory investigations revealed hemoglobin of 11 g%, total leukocyte count of 16,800 cells/μL with neutrophilia, and ESR of 80 mm at the end of the first hour. She was negative for HIV, HBsAg and VDRL. Sputum for acid-fast bacilli (AFB) was also negative (three samples).

**Figure 1:**
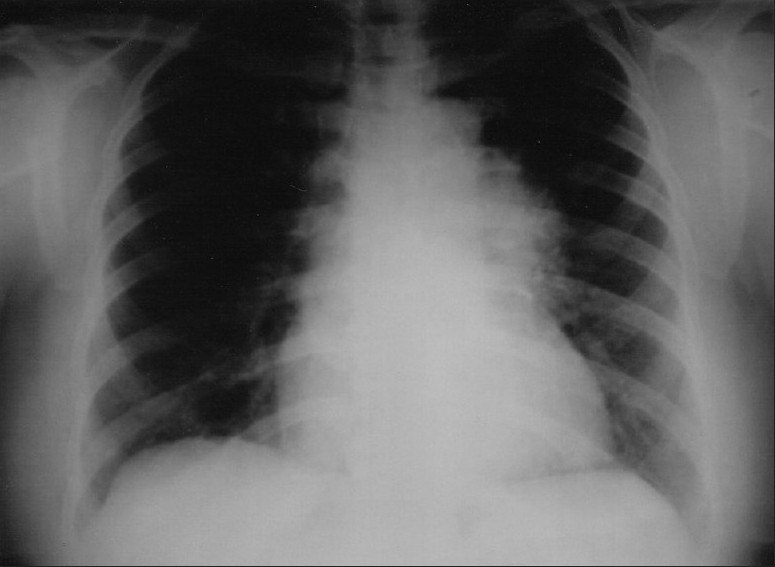
X-ray chest, PA view, showing non-homogenous mass lesion near the left hilum

**Figure 2:**
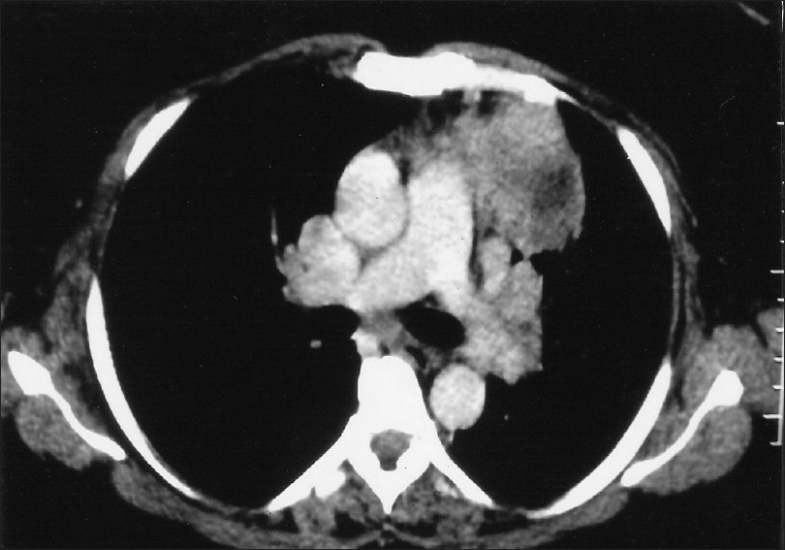
Computed tomography scan of the chest showing heterogeneously enhancing soft tissue density in the left upper lobe

Chest radiograph revealed a non-homogenous mass lesion near the left hilum [Figure 1]. Computed tomography (CT) scan of the thorax (plain and contrast) showed evidence of a heterogeneously enhancing soft tissue mass in the left upper lobe region, approximately measuring 8.2 × 5.4 cm [Figure 2]. CT of the abdomen was normal. Fiber-optic bronchoscopy showed an intrabronchial mass with almost complete occlusion of the left upper lobe bronchial lumen. Fine-needle aspiration cytology (FNAC), bronchoalveolar lavage (BAL) and endobronchial brush biopsy (EBB) were inconclusive. Endobronchial biopsy from the mass showed inflammatory lesion. No atypical cells were present. BAL and EBB were negative for acid-fast bacilli.

**Figure 3:**
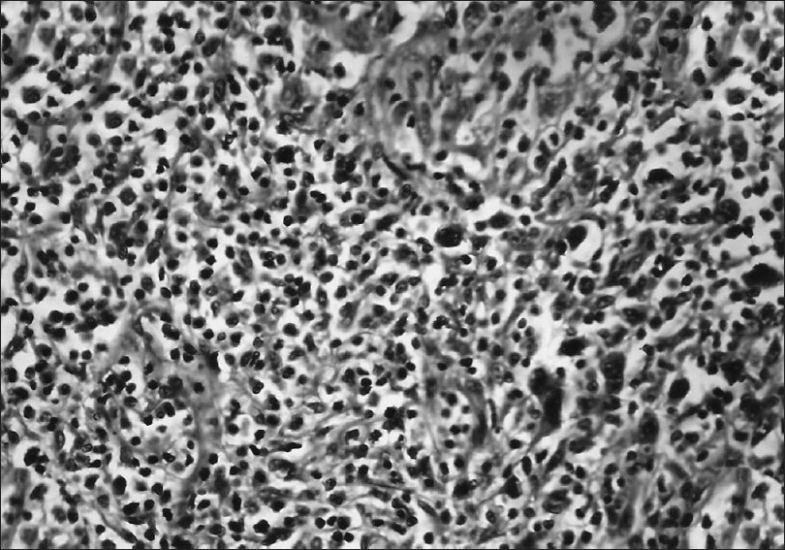
Photomicrograph showing large pleomorphic cells admixed with mixed inflammatory cells (H and E, ×200)

**Figure 4:**
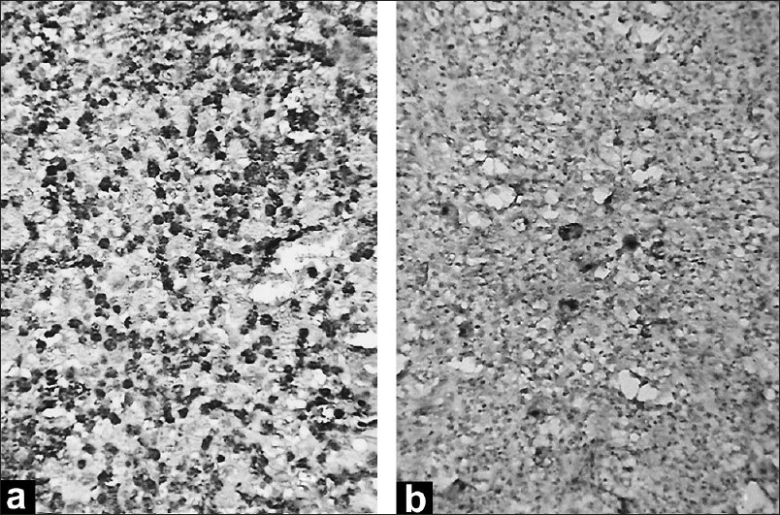
Photomicrograph showing large cells immunopositive for (a) CD15; and (b) CD30; (immunohistochemistry for CD15 and CD30 ×400)

As the diagnosis was inconclusive, the patient was taken for open thoracotomy. It showed a large mass in the left upper lobe and lingual lobe with extension towards the hilum and encasement of the large vessels. Partial excision of the mass was done as complete excision was not possible due to the entrapment of the greater vessels. The resected specimen examination revealed the mediastinal surface showing prominent bronchioles containing grayish white friable mass measuring 6.4 × 4.5 cm. Sections from the mass revealed numerous large cells admixed with neutrophils, eosinophils, and lymphocytes. The large cells had moderate amount of eosinophilic cytoplasm and large pleomorphic nuclei [Figure 3]. Few of these cells showed binucleation and multinucleation. Foci of necrosis were present. The adjacent lung tissue showed features of organizing pneumonia. A provisional diagnosis of anaplastic large cell non-Hodgkin's lymphoma was considered. On immunohistochemistry, the large cells were positive for CD15 and CD30 [Figure 4]. CD3 was detected in the interstitial infiltrate; and CD45, diffusely. A final diagnosis of primary pulmonary Hodgkin's disease (PPHD) was made. The patient was started on chemotherapy consisting of adriamycin (25 mg/m^2^), bleomycin (10 units/m^2^), vinblastine (6 mg/m^2^) and dacarbacin (375 mg/m^2^) every four weeks for eight cycles. Following chemotherapy, the lung mass regressed, and the patient is free of the symptoms for the last one year and is performing all the household activities. Follow-up CT scan of the thorax showed almost complete regression of the mass.

## DISCUSSION

L'Hoste and associates[2] have proposed the following criteria for diagnosing primary pulmonary lymphoma: (i) involvement of the lung, lobar or primary bronchus, with or without mediastinal involvement; and (ii) no evidence of extra-thoracic lymphoma at the time of diagnosis or for three months thereafter. PPHD accounts for 3.6% of extranodal lymphomas,[3] the peak incidence being in the sixth or seventh decade of life. Radin[1] reviewed 60 published cases of Hodgkin's lymphoma and has reported that lymphoma affects women more frequently and shows bimodal age distribution (<35 years and >60 years). Only a few cases of primary Hodgkin's disease of the lung have been reported in the literature.

Hodgkin's disease presents as a solitary mass, cavitatory lesion or diffuse involvement of the lungs. Presentation of Hodgkin's disease as an endobronchial lesion is very uncommon. Due to respiratory symptoms, which can be predominant in these cases, chest physicians may be confronted with the diagnosis of this condition. The possible mechanism responsible for endobronchial disease is either by direct bronchial invasion or by hematogenous dissemination. Moolten[4] reported two cases of Hodgkin's lymphoma with airway involvement, in 1934; and Tredaniel *et al.,*[5] reviewed the endobronchial presentation of Hodgkin's disease, and they could get only nine cases from the literature. Dyspnea and coughing were the most frequent symptoms. Our patient had endobronchial extension of the disease with intraluminal growth on fiber-optic bronchoscopy. It has been proposed that to be recognized as an endobronchial presentation of Hodgkin's disease, the case has to fulfill the following criteria[5] at the time of initial diagnosis of the disease: (i) histological features of Hodgkin's disease (whatever the site of biopsy) and (ii) bronchoscopic visualization of an endobronchial tumor.

Clinically, the differential diagnosis includes tuberculosis, fungal granulomas, and lung carcinoma. As an endobronchial extension in Hodgkin's disease is rare, it is often missed. Bronchoscopy and bronchial cytology are unrevealing in most of the cases. Boshnakova *et al.*[6] have also reported that bronchoscopic biopsies were inconclusive in their two cases with endobronchial involvement, due to lack of Reed-Sternberg cells.

Histologically, the differential diagnosis includes large B cell lymphoma, anaplastic large cell lymphoma, large cell carcinoma, and melanoma. If extensive necrosis and granulomas are seen, the presence of Reed-Sternberg cells helps to exclude infectious granulomas and granulomatosis. Undifferentiated carcinomas may show the presence of HRS-like cells, but neutrophils are frequently seen and eosinophils are seldom present. Some forms of T cell lymphomas may simulate Hodgkin's disease by showing extreme degree of pleomorphism and a background of reactive inflammatory cells. Immunohistochemistry is required in such cases for a confirmatory diagnosis.[7] The large mononuclear cells of large B cell lymphoma are positive for CD20 rather than CD30. In anaplastic large cell lymphoma, the neoplastic cells usually exhibit a T cell phenotype (CD3^+^, CD30^+^, EMA and often Alk 1 kinase^+^). Large cell carcinomas are immunopositive for keratins, CD30-, and melanoma cells are S-100^+^. Endobronchial Hodgkin's disease may also be mistaken for small cell carcinoma.[8] In our case, presence of large pleomorphic cells with single-to-multiple nuclei admixed with neutrophils, eosinophils, and lymphocytes; and absence of classical Reed-Sternberg giant cells (HRS) made us consider the diagnosis of anaplastic large cell lymphoma. However, the large cells were positive for CD15 and CD30, thus clinching the diagnosis of Hodgkin's disease. Our case fits in stage I_E_ according to Ann Arbor pulmonary lymphoma staging system.

In Hodgkin's disease, careful staging and optimal treatment can lead to cure for approximately 75% of patients. The factors that correlate with poor prognosis include old age, “B” symptoms, bilateral disease, multilobar involvement, penetration of pleura, and cavitations.[2] Therefore, primary endobronchial Hodgkin's disease, which is most often associated with bulky mediastinum, is now treated by combined-modality therapy. Our patient responded well with combination chemotherapy given every four weeks for eight cycles, and repeat CT scan of thorax showed good resolution of the mass; and she is free of symptoms for the last one year.

The prognosis for patients with PPHD is generally good. Cordier *et al.*[9] have reported a five-year survival rate of 94% for low-grade PPHD, and a median survival of three years for high-grade disease. Patients can have poor outcomes when the disease presents with extensive extra-thoracic disease.
